# Transcatheter Electrosurgery in the Asia-Pacific: Implementation and Innovations

**DOI:** 10.1016/j.jacasi.2025.10.025

**Published:** 2026-01-06

**Authors:** Chun-Ka Wong, Karl Poon, Kent Chak-Yu So, Wei-Hsian Yin, Yung-Tsai Lee, Mann Chandavimol, Kwong-Yue Eric Chan, Daniel Tai-Leung Chan, Leo Kar Lok Lai, Kentaro Hayashida, Duk-Woo Park, Yohei Ohno, Kay-Woon Ho, Jung-Sun Kim, Tsuyoshi Kaneko, Vinayak N. Bapat, Alan Yeung, Nattawut Wongpraparut, Ho-On Alston Conrad Chiu, Ching-Wei Lee, Gilbert H.L. Tang, Jian'an Wang, Jaffar M. Khan, Simon Cheung-Chi Lam

**Affiliations:** a Cardiology Division, Department of Medicine, School of Clinical Medicine, Li Ka Shing Faculty of Medicine, The University of Hong Kong, Hong Kong SAR; b Cardiology Division, Department of Medicine, Queen Mary Hospital, Hong Kong SAR; c Cambridge Stem Cell Institute, University of Cambridge, Cambridge, United Kingdom; d University of Queensland, Brisbane, Australia; e The Prince Charles Hospital, Metro North Health, Brisbane, Australia; f Division of Cardiology, Department of Medicine and Therapeutics, The Chinese University of Hong Kong, Hong Kong SAR; g Li Ka Shing Institutes of Health Science, Prince of Wales Hospital, The Chinese University of Hong Kong, Hong Kong SAR; h Heart Center, Cheng Hsin General Hospital, Taipei, Taiwan; i Faculty of Medicine, School of Medicine, National Yang Ming Chiao Tung University, Hsinchu, Taiwan; j Department of Medicine, Ramathibodi Hospital, Mahidol University, Bangkok, Thailand; k Cardiac Medical Unit, Grantham Hospital, Hong Kong SAR; l Department of Cardiothoracic Surgery, Queen Mary Hospital, Hong Kong SAR; m Department of Surgery, School of Clinical Medicine, Li Ka Shing Faculty of Medicine, The University of Hong Kong, Hong Kong SAR; n Division of Cardiology, Department of Medicine and Therapeutics, Prince of Wales Hospital, Chinese University of Hong Kong, Hong Kong SAR; o Department of Cardiology, Keio University School of Medicine, Tokyo, Japan; p Division of Cardiology, Asan Medical Center, University of Ulsan College of Medicine, Seoul, Korea; q Department of Cardiology, Tokai University School of Medicine, Isehara, Japan; r Department of Cardiology, National Heart Centre Singapore, Singapore; s Division of Cardiology, Severance Cardiovascular Hospital, Yonsei University College of Medicine, Seoul, Korea; t Department of Surgery, Washington University in St Louis, St Louis, Missouri, USA; u Department of Cardiac Surgery, Minneapolis Heart Institute at Abbott Northwestern Hospital, Minneapolis, Minnesota, USA; v Division of Cardiovascular Medicine, Department of Medicine, Stanford University, Stanford, California, USA; w Department of Medicine, Siriraj Hospital, Mahidol University, Bangkok, Thailand; x Cardiovascular Center, Taipei Veterans General Hospital, Taipei, Taiwan; y Department of Cardiovascular Surgery, Mount Sinai Health System, New York, New York, USA; z Department of Cardiology, The Second Affiliated Hospital, Zhejiang University School of Medicine, Hangzhou, Zhejiang, China; aa Cardiovascular Key Laboratory of Zhejiang Province, Hangzhou, Zhejiang, China; bb State Key Laboratory of Transvascular Implantation Devices, Hangzhou, Zhejiang, China; cc St Francis Hospital and Heart Center, Roslyn, New York, USA

**Keywords:** transcatheter aortic valve replacement, transcatheter electrosurgery, transcatheter mitral valve replacement

## Abstract

Transcatheter electrosurgery involves the precise application of high-frequency electrical currents to modify or perforate cardiac structures during structural heart interventions. Adoption of these techniques in the Asia-Pacific region has grown significantly alongside the expansion of structural heart programs. Techniques such as Bioprosthetic or Native Aortic Scallop Intentional Laceration to Prevent Coronary Artery Obstruction (BASILICA), Undermining Iatrogenic Coronary Obstruction With Radiofrequency Needle (UNICORN), Laceration of the Anterior Mitral Leaflet to Prevent Outflow Obstruction (LAMPOON), and Balloon Assisted Translocation of the Mitral Anterior Leaflet to Prevent Left Ventricular Outflow Obstruction (BATMAN) have been employed by regional interventionalists to lacerate native or prosthetic heart valves before transcatheter valve implantation, thereby mitigating the risks of coronary or left ventricular outflow tract obstruction. In addition, transcaval access has facilitated transcatheter aortic valve replacement in patients with limited iliofemoral vascular access. This narrative review, authored by the Asia-Pacific Electrosurgery Working Group, explores the evolution of transcatheter electrosurgery, highlighting regional adoption, variations, and innovative contributions. Furthermore, it discusses prevailing challenges and future directions, emphasizing the importance of standardized training and collaborative innovation efforts.

Transcatheter electrosurgery refers to the use of precisely delivered, high-frequency electrical current to modify or perforate cardiac structures during structural heart interventions.^1^^,^^2^ Initially, these techniques were developed primarily to lacerate native or prosthetic heart valves before the implantation of prosthetic valves, aiming to reduce the risk of coronary or left ventricular outflow tract (LVOT) obstruction. The Bioprosthetic or Native Aortic Scallop Intentional Laceration to Prevent Coronary Artery Obstruction (BASILICA) procedure was introduced to prevent coronary artery obstruction during transcatheter aortic valve replacement (TAVR) by intentionally lacerating the aortic leaflet that could otherwise block the coronary ostia.^3^^,^^4^ Similarly, the Laceration of the Anterior Mitral Leaflet to Prevent Outflow Obstruction (LAMPOON) technique was designed to prevent LVOT obstruction during transcatheter mitral valve replacement (TMVR) by lacerating the anterior mitral leaflet.^5^^,^^6^ Other transcatheter electrosurgical techniques used to address different anatomies include transcaval access,^7^ removal of transcatheter edge-to-edge repair clips,^8^ and the treatment of hypertrophic cardiomyopathy.^9^^,^^10^

In the Asia-Pacific region, transcatheter electrosurgery is particularly relevant because a significant proportion of patients present with high-risk anatomical features. There is a higher prevalence of patients with small aortic root,^11^^,^^12^ which could lead to higher coronary obstruction risk for both native valve and valve-in-valve TAVR. It is also not uncommon to encounter cases in which both coronary arteries are at risk of obstruction, in which case bilateral leaflet modification may be required (Figure 1). In addition, Asian patients often have smaller left ventricular cavity sizes,^13^ and in certain patients this may heighten the risk of LVOT obstruction following TMVR, requiring anterior mitral valve leaflet (AMVL) modification before valve implantation. Other factors driving demand for transcatheter electrosurgery in the Asia-Pacific region include the steadily rising number of TAVR procedures,^14^, ^15^, ^16^, ^17^ which is expected to lead to a surge in valve-in-valve interventions.^18^ Furthermore, with the generally high life expectancy in the Asian population,^19^ there is increasing demand for transcatheter therapies across native, valve-in-ring, and valve-in-valve settings.

**Figure 1:**
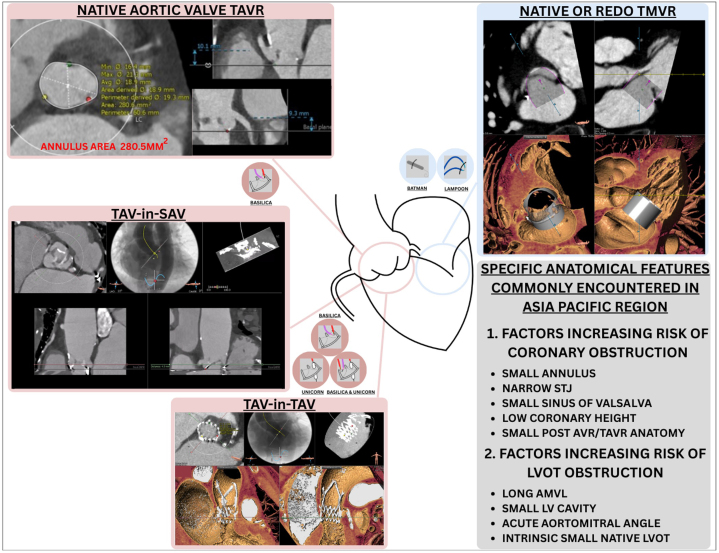
Complex Anatomical Features in Patients From the Asia-Pacific Regions High-risk features such as a small aortic root among the Asian-Pacific population may increase the risk of transcatheter heart valve implantation and warrants the use of leaflet modification techniques. AMVL = anterior mitral valve leaflet; AVR = aortic valve replacement; BASILICA = Bioprosthetic or Native Aortic Scallop Intentional Laceration to Prevent Coronary Artery Obstruction; BATMAN = Balloon Assisted Translocation of the Mitral Anterior Leaflet to Prevent Left Ventricular Outflow Obstruction; LAMPOON = Laceration of the Anterior Mitral Leaflet to Prevent Outflow Obstruction; LV = left ventricle; LVOT = left ventricular outflow tract; STJ = sino-tubular junction; TAVR = transcatheter aortic valve replacement; TAV-in-SAV = transcatheter aortic valve replacement in surgical aortic valve; TAV-in-TAV = transcatheter aortic valve replacement in transcatheter aortic valve; TMVR = transcatheter mitral valve replacement; UNICORN = Undermining Iatrogenic Coronary Obstruction With Radiofrequency Needle.

In recent years, transcatheter electrosurgery has been gradually adopted in the Asia-Pacific region with outcome comparable to other regions.^20^, ^21^, ^22^, ^23^ However, adoption of these advanced techniques is challenging in the region, as there is a relatively small number of experienced operators available to offer proctorship. In addition, some dedicated tools for transcatheter electrosurgery are not readily available in the region. To overcome the unique anatomical challenges and resource limitations, innovative approaches have emerged in the region, including the development of Undermining Iatrogenic Coronary Obstruction With Radiofrequency Needle (UNICORN) and other novel techniques.^20^^,^^24^^,^^25^

In this narrative review article written by the Asia-Pacific Electrosurgery Working Group, we explore the evolution of transcatheter electrosurgery and discuss the Asia-Pacific adoption of these techniques, highlighting regional variations and contributions to innovation. Finally, we address the challenges and future directions in the field, considering the need for standardized training and collaborative efforts in innovation (Central Illustration).

**Central Illustration:**
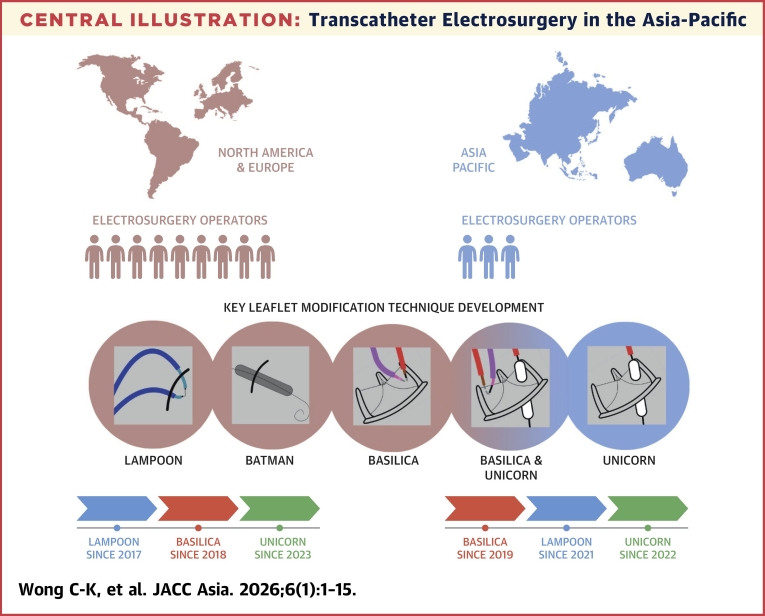
Transcatheter Electrosurgery in the Asia-Pacific Transcatheter electrosurgical techniques such as BASILICA (Bioprosthetic or Native Aortic Scallop Intentional Laceration to Prevent Coronary Artery Obstruction), LAMPOON (Laceration of the Anterior Mitral Leaflet to Prevent Outflow Obstruction), and BATMAN (Balloon Assisted Translocation of the Mitral Anterior Leaflet to Prevent Left Ventricular Outflow Obstruction) were pioneered in North America and Europe since 2017. At present, there are relatively fewer operations with experience in electrosurgery in the Asia-Pacific region. Nevertheless, case volume has been steadily growing and innovative techniques such as UNICORN (Undermining Iatrogenic Coronary Obstruction With Radiofrequency Needle) have emerged from the region.

## Aortic Valve

The case volume of TAVR has been rising steadily in the Asia-Pacific region in the past decade. For instance, the volume of TAVR rose from 293 in 2017 to 7,357 in 2021 in China.^14^, ^15^, ^16^, ^17^ Despite achieving excellent results in most patients, coronary obstruction may occur in approximately 0.8% patients who received TAVR,^26^ with the risk increasing 3- to 4-fold in valve-in-valve procedures.^27^ Coronary obstruction risk factors include low coronary ostial height (<10 mm), narrow sinus of Valsalva with virtual transcatheter valve-to-coronary ostium distance <4 mm, heavy leaflet calcification, and valve-in-valve procedures using externally mounted or stentless surgical bioprostheses, which could lead to higher coronary obstruction risk for native valve and valve-in-valve TAVR.^14^, ^15^, ^16^, ^17^ The higher prevalence of patients with small aortic root in the region prompted adoption of BASILICA and invention of the UNICORN technique.^11^^,^^12^^,^^20^

### BASILICA

**Figure 2:**
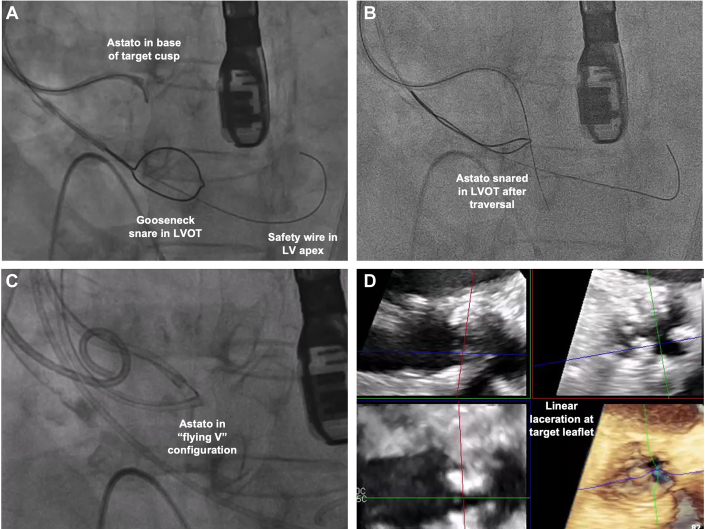
BASILICA Technique (A) Astato guidewire directed toward the base of the target leaflet. Gooseneck snare in the LVOT. Safety wire in the LV apex. (B) After traversal of the target leaflet, Astato guidewire is snared in the LVOT. (C) A "flying V" configuration is created in the Astato wire by denuding and kinking a segment near the end of a wire converter, such as PiggyBack wire converter (Teleflex) or 0.035-inch microcatheter. (D) Echocardiography showing linear laceration at the target leaflet. Abbreviations as in Figure 1.

The BASILICA procedure was first described in 2018 as a procedure to reduce coronary obstruction risk by transcatheter electrosurgical laceration of the aortic valve leaflet^4^ (Table 1). An LVOT catheter is advanced through the aortic valve to deploy a snare below the valve level. An 0.018-inch guidewire is placed in the left ventricle as anchor. Simultaneously, an aortic catheter equipped with a 0.014-inch Astato XS 20 guidewire (Asahi Intecc Medical), loaded within a wire connector such as PiggyBack wire converter (Teleflex) or 0.035-inch microcatheter, is directed toward the base of the target leaflet under both transesophageal echocardiography and fluoroscopic guidance (Figure 2A). The back of the Astato wire is denuded using a scalpel blade and then connected to an electrocautery pencil using forceps. Traversal of the aortic valve with the Astato wire is achieved by applying brief electrocautery in “pure cut” mode (30–50 W), after which it is snared (Figure 2B). A “flying V” configuration is created in the Astato wire by denuding and kinking a segment near the end of the wire converter (Figure 2C). This assembly is repositioned at the target leaflet and secured with torque devices. During laceration, 5% dextrose is injected through both catheters to concentrate the electrical current, while "pure cut" energy (50–70 W) is applied to the electrified wire and gentle tension is maintained on both catheters to cleanly divide the leaflet without mechanical avulsion (Figure 2D). The procedure concludes with standard TAVR deployment following successful leaflet modification. The safety and efficacy of the BASILICA procedure have been demonstrated in prospective trials as well as in real-world registries^3^^,^^28^, ^29^, ^30^, ^31^ (Table 2). Potential complications include residual coronary obstruction risk, intercurrent aortic regurgitation, and vascular and cardiac injuries.

**Table 1:** Common Tools for BASILICA and UNICORN

BASILICA
Electrosurgery generator, pencil, and forceps
GORE DRYSEAL Flex Introducer Sheath (Gore Medical)
Perclose ProGlide (Abbott)
SENTINEL Cerebral Protection System (Boston Scientific)
BHW (Abbott)/ V-14 ControlWire (Boston Scientific) 0.014-inch 300cm
V-18 ControlWire 0.014-inch 300 cm (Boston Scientific)
Astato XS 20 0.014-inch 300-cm wire (Asahi)
Piggyback Wire Converter (Teleflex)/ Finecross (Terumo)
Guiding catheter: Left - 6F AL2/3; Right - 6F MPA/ JR4
Snare catheter - gooseneck 20/25 mm
60 mL Luer lock syringes for Dextrose 5% flush
Torquers - 0.014-inch and 0.035-inch
Scalpel, needle holders
UNICORN
RFP-100A RF Puncture Generator (Boston Scientific)
Edwards eSheath+ (Edwards Lifesciences) (or equivalent)
6F Sheath (contralateral access)
Perclose ProGlide (Abbott)
SENTINEL Cerebral Protection System (Boston Scientific)
BHW (Abbott)/ V-14 ControlWire (Boston Scientific) 0.014-inch 300 cm
VersaCross J-tip RF wire (Boston Scientific)
NaviCross Angled-tip Support Catheter 4F 135 cm (Terumo)
Guiding catheter: Left - 7F AL1, AL2 (100 cm); Right - 7F AL1, MP (100 cm)
NC coronary balloon - 3.0 × 15-20 mm
Peripheral balloon - 10 × 40 mm
S3UR transcatheter heart valve (Edwards Lifesciences)

BASILICA = Bioprosthetic or Native Aortic Scallop Intentional Laceration to Prevent Coronary Artery Obstruction; RF = radiofrequency; UNICORN = Undermining Iatrogenic Coronary Obstruction With Radiofrequency Needle.

**Table 2:** BASILICA Outcome

Trial/ Registry	No. of Patients	Traversal Success, %	Laceration Success, %	Technical/Procedural/ Clinical Success, %	Coronary Obstruction or Intervention, %	Ref. #
BASILICA FIM	7	100	100	100	0	^4^
BASILICA IDE prospective trial	30	93.0		93.0	0	^3^
International BASILICA registry	214	94.9	94.4	86.9	4.7	^29^
EURO-BASILICA registry	76	98.8^a^	88.2^a^	97.7^a^	9.4	^30^
Hamburg single-center series	15	86.7		86.7	6.7	^28^
Chimney stenting vs BASILICA multicenter observational registry	97	N/A	N/A	Clinical success 96.9	8.2	^31^

FIM = first-in-man; IDE = investigational device exemption; N/A = not applicable; other abbreviations as in Table 1.

a Percentage calculated by number of leaflets attempted.

Since 2019, the procedure and its variants, such as balloon-assisted BASILICA, have been increasingly adopted in the Asia-Pacific region.^32^, ^33^, ^34^ At the time of writing, dedicated guidewire systems for performing BASILICA such as the TELLTALE (Transmural Systems) and devices engineered to mechanically split the aortic valve leaflet in a manner similar to BASILICA, such as the ShortCut Device (Pi-Cardia),^35^ are not yet available in the Asia-Pacific region.

### UNICORN

**Figure 3:**
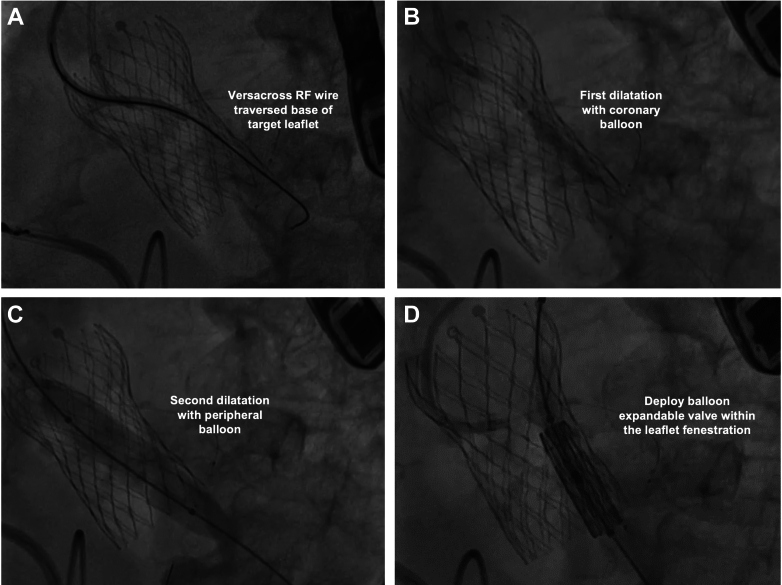
UNICORN Technique With Balloon-Expandable Valve (A) VersaCross RF wire is used to traverse the base of the target leaflet. (B) First balloon dilatation with coronary balloon. (C) Second dilatation with peripheral balloon. Target leaflet is not lacerated after this step. (D) Balloon-expandable valve is deployed within the newly created fenestration. RF = radiofrequency; other abbreviation as in Figure 1.

The UNICORN procedure is an alternative method developed in Hong Kong for alleviating coronary obstruction risk during valve-in-valve TAVR^20^^,^^24^ (Table 1). In this technique, a telescoping system comprising a 7F Amplatz Left-1 guide catheter (Cordis), an angled-tip 135-cm Navicross 0.035-inch microcatheter (Terumo), and a 0.035-inch J-tip VersaCross radiofrequency (RF) wire (Boston Scientific) is first positioned at the base of the target leaflet. Thereafter, 1 to 2 seconds of RF pulse is applied to allow the RF wire to traverse the leaflet (Figure 3A), targeting the nadir of the cusp. Once the Navicross catheter has crossed the leaflet, a 300-cm coronary wire is inserted into the left ventricle. An alternative traversal strategy adopted by some interventionalists is to use of electrified 0.014-inch or 0.035-inch wire, such as the Astato wire, instead of the VersaCross RF wire.^36^, ^37^, ^38^, ^39^

Subsequently, 2-step sequential dilatations of the created fenestration is performed. First dilatation is performed using a 3- to 4-mm noncompliant coronary balloon (Figure 3B). Following the initial fenestration, the coronary wire is exchanged for a pre-shaped stiff wire. Second balloon dilatation, often with double tap and higher than nominal pressure to ensure adequate expansion, is then performed using a 10- to 12-mm peripheral balloon to further enlarge the fenestration so that the transcatheter heart valve (THV) can pass (Figure 3C). A balloon-expandable valve is inserted into the fenestration (Figure 3D). As the THV inflates, the target aortic leaflet is simultaneously lacerated and pushed away from the at-risk coronary ostium.

When the UNICORN technique is used with balloon-expandable valves, the target leaflet is opened concurrently at the same time of intraleaflet THV deployment, within the created fenestration before any unintentional leaflet lacerations.^20^ The advantage of this approach is that as the new THV closely abuts the annulus at the at-risk region, it is theoretically less likely for the newly lacerated leaflet to occupy the space between the THV and the annulus, potentially offering better protection to the coronary ostium. Basal leaflet transversal is key to success for subsequent most-optimal and predictable leaflet clearance from coronary ostia. Registry data to be published from Asia-Pacific and other regions will provide further information regarding the technique’s safety and efficacy. Potential complications include residual coronary obstruction risk, intercurrent aortic regurgitation if the leaflet is prematurely lacerated before intraleaflet valvular implantation, valve-leaflet interaction, and vascular and cardiac injuries.^40^

In the past few years, when the technique is used with a self-expandable valve (SEV), operators often use an oversized balloon to lacerate the leaflet before valve deployment.^36^, ^37^, ^38^ Recently, a novel recrossing technique has been described that facilitates near-simultaneous leaflet laceration and SEV deployment.^25^ It is important to recognize that there is currently a lack of published data demonstrating the safety of applying UNICORN in native valves.

For the preceding aortic leaflet modifications techniques—BASICILA, UNICORN, or sometimes hybrid approaches—concomitant coronary protection with the use of coronary wire, guide extensions, or coronary balloons with or without coronary stents also would be considered depending on intrinsic coronary obstruction risks due to individual anatomical factors.^41^

## Mitral Valve

In the Asia-Pacific region, TMVR is still in its early stages. Native valve TMVR has been extremely limited because devices dedicated to this procedure, which are already approved for clinical use in Europe,^42^ as well as investigational products under prospective study in North America, are not available in the region.^43^, ^44^, ^45^, ^46^ Valve-in-mitral annular calcification procedures have been infrequently performed,^47^ whereas valve-in-ring and valve-in-valve TMVR procedures have received regulatory approval or reimbursement in an increasing number of countries over the past 2 to 3 years.

As the number of TMVR procedures gradually increases, there is a growing need to develop strategies to mitigate the risk of LVOT obstruction. After TMVR implantation, the AMVL may be displaced toward the LVOT, causing obstruction. Computed tomography analysis has identified several predictors for LVOT obstruction, including a small neo LVOT area (<170 to 190 mm^2^), an acute aorto-mitral angle, a long AMVL, and septal hypertrophy.

In the Asia-Pacific region, the primary strategy to reduce the risk of LVOT obstruction has been the laceration of the AMVL using the tip-to-base LAMPOON technique (Table 3).^21^ There also have been initial experiences with the Balloon Assisted Translocation of the Mitral Anterior Leaflet to Prevent Left Ventricular Outflow Obstruction (BATMAN) technique for mechanical AMVL laceration.^48^ Meanwhile, septal reduction strategies have been limited to alcohol septal ablation, as experience with the SEptal Scoring Along Midline Endocardium (SESAME) technique is limited in the region.^9^^,^^10^

**Table 3:** Common Tools for LAMPOON

Electrosurgery generator, pencil, and forceps
Edwards eSheath+ Introducer Set (Edwards Lifesciences) for S3UR (or equivalent)
Perclose ProGlide (Abbott) (or hemostatic suture)
SENTINEL Cerebral Protection System (Boston Scientific)
BHW (Abbott)/V-14 ControlWire (Boston Scientific) 0.014-inch 300 cm
Astato XS 20 300 cm 0.014-inch wire
Piggyback Wire Converter (Teleflex)/Finecross (Terumo)
Transseptal Puncture System, such as VersaCross (Boston Scientific), BRK Transseptal Needle (Abbott), and SL1 Transseptal Sheath (Abbott)
Guiding Catheter: 6F JR4 (for transversal); 6F EBU 3/JL 3.5 (for LVOT snare)
Snare catheter - gooseneck/EN snare (Merit Medical System)
6F Balloon-wedge end-hole catheter
60-mL Luer lock syringes for Dextrose 5% flush
Torquers – 0.014-inch and 0.035-inch
Scalpel, needle holders
Intra-aortic balloon pump

LAMPOON = Laceration of the Anterior Mitral Leaflet to Prevent Outflow Obstruction; LVOT = left ventricular outflow tract.

### LAMPOON

LAMPOON was first conceived in 2016 in recognition of the high risk of LVOT obstruction for mitral valve-in-ring and valve-in-mitral annular calcification procedures, as well as native mitral valve TMVR.^5^ In mitral valve-in-valve procedures, the risk of LVOT obstruction is inherently lower, as the native AMVL would have been resected during a previous operation.^49^^,^^50^

The original base-to-tip LAMPOON was a bi-arterial retrograde approach involving 2 guide catheters through the aortic valve, one on the ventricular side of the mitral valve and one on the atrial side through the mitral valve. The ventricular guide houses the Astato wire for traversal of the AMVL. This wire is then snared in the left atrium, establishing a rail for “flying V” laceration in a manner similar to BASILICA.^51^ A transseptal steerable catheter in the left atrium, establishing a rail with the atrial guide, can improve snaring and readiness to deploy a transseptal mitral valve prosthesis.^52^

**Figure 4:**
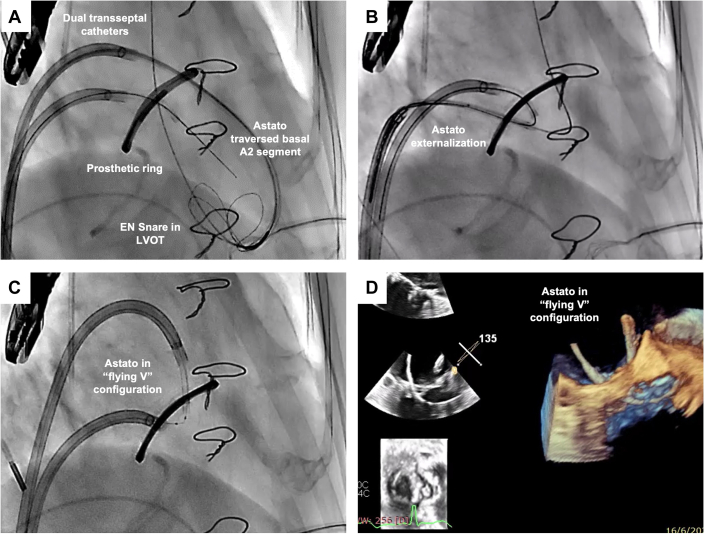
Antegrade Base-to-Tip LAMPOON Technique (A) Two transseptal steerable sheaths are placed in the left atrium, one for delivering Astato wire for traversing the A2 segment of the anterior mitral valve leaflet; and another one for positioning the EN Snare in the LVOT. (B) After leaflet traversal, the Astato is snared and externalized. (C) Astato wire in “flying V” configuration is used to lacerate the anterior mitral valve leaflet. (D) “Flying V” shown on transesophageal echocardiography. Abbreviations as in Figure 1.

Antegrade base-to-tip LAMPOON is a purely transseptal antegrade technique and simplifies the LAMPOON procedure.^53^ Two transseptal steerable sheaths are placed in the left atrium, one for traversal through the AMVL, and one with a JL3.5 guide catheter positioned in the LVOT with an EN Snare to catch the traversed wire. This then allows laceration of the AMVL by pulling atrially (Figure 4).

For complete mitral annuloplasty rings, a further simplification was the tip-to-base LAMPOON technique.^23^^,^^53^^,^^54^ This involves transseptal access, with a balloon-tipped catheter floated into the ascending aorta. A retrograde aortic guide catheter can then be used to snare the Astato wire. The electrified “flying V” in this wire can lacerate the AMVL from the tip to the base of the mitral valve without requiring leaflet traversal due to the hard stop at the ring level. It is essential to ensure the ventricular guide remains below the aortic leaflet to avoid leaflet injury or torrential regurgitation. The safety and efficacy of the LAMPOON procedure have been demonstrated in prospective trials as well as in real-world registries^5^^,^^6^^,^^21^^,^^23^^,^^53^^,^^55^ (Table 4). Potential complications mainly include residual LVOT obstruction risk and injury to vascular and cardiac structure including the aortic valve.

**Table 4:** LAMPOON Outcome

Trial/Registry	No. of Patients	Approach	Traversal Success, %	Laceration Success, %	Technical/ Procedural/Clinical Success, %	LVOT Obstruction, or Rescue LAMPOON, %	Ref. #
LAMPOON FIH	5	Retrograde Base-to-tip	100	100	100	0	^5^
LAMPOON IDE study	30	Retrograde Base-to-tip	100	100	73	3	^6^^,^^55^
Antegrade LAMPOON	8	Antegrade Base-to-tip	100	100	100	9.4	^53^
Tip-to-base LAMPOON	21	Antegrade Tip-to-base	100	100	100	0	^23^
Rendezvous LAMPOON	13	Antegrade Tip-to-base	100	100	100	0	^21^

FIH = first-in-human; other abbreviations as in Tables 2 and 3.

Since 2021, the tip-to-base LAMPOON procedure has been performed in the Asia-Pacific region.^21^^,^^56^ A group in Taipei proposed a modified "rendezvous" technique: rather than using a balloon-tipped catheter in the aorta, a slippery wire was floated into the ascending aorta, taking care not to entangle the wire in the mitral chordae. A multipurpose 5-Fr catheter would then rendezvous from the left atrial inside the 7-Fr JR guide catheter in the ascending aorta over the slippery 300-cm wire.^21^ Currently, devices for mechanically splitting the AMVL are not yet available in the Asia-Pacific region.^57^^,^^58^

### BATMAN

The BATMAN technique is an alternative technique for lacerating the AMVL to reduce the risk of LVOT obstruction following TMVR. When the technique was first described, it was performed as a transapical procedure.^59^^,^^60^ Subsequently, the BATMAN procedure has been successfully performed using a percutaneous transseptal approach in Asia-Pacific and other regions^39^^,^^48^^,^^60^, ^61^, ^62^, ^63^, ^64^, ^65^, ^66^, ^67^ (Table 5).

**Table 5:** Common Tools for BATMAN

No. of Cases	MCS	Transseptal	AMVL Traversal	1st Dilatation	2nd Dilatation	Septal Dilatation	Leaflet Laceration Before Valve Deployment	Buddy Wire	Ref. #
1	Cardio-pulmonary bypass	N/A (Transapical)	18G pericardiocentesis needle	N/A	20 mm	N/A (Transapical)	No	No	^59^
3	Cardio-pulmonary bypass	N/A (Transapical)	18G pericardiocentesis needle	N/A	20 mm	N/A (Transapical)	No	No	^60^
1	No	Not specified	0.035-inch electrified wire	6 mm	12 mm	Not specified	No	No	^61^
3	±IABP	Not specified	0.035-inch electrified wire	No		14-F^a^	No	Yes	^62^
1	No	Not specified	0.014-inch electrified wire	4 mm	12 mm	Not specified	No	No	^63^
1	No	0.035-inch electrified wire		4 mm	14 mm 26 mm	Not specified	Yes	No	^66^
1	IABP	Transseptal needle	0.014-inch electrified wire	4 mm		14 mm^a^	No	No	^64^
1	IABP	VersaCross RF wire^b^		6 mm	12 mm	14 mm	No	No	^65^
2	No	VersaCross RF wire^b^		6 mm		12 mm^a^	No	No	
1	IABP	VersaCross RF wire^b^		3 mm		14 mm^a^	No	No	^48^
4	No	0.035-inch electrified wire^b^		4 mm		12 mm^a^	No	No	^39^
2	No	0.035-inch electrified wire	0.014-inch electrified wire	4 mm		12 mm^a^	No	No	
2	No	Not specified	0.014-inch electrified wire	3.5-4 mm		14 mm	No	No	^67^

AMVL = anterior mitral valve leaflet; BATMAN = Balloon Assisted Translocation of the Mitral Anterior Leaflet to Prevent Left Ventricular Outflow Obstruction; IABP = intra-aortic balloon pump; MCS = mechanical circulatory support; N/A = not applicable.

a The same tool was used for dilating both the septum and AMVL in these cases.

b The same tool was used for both transseptal puncture and AMVL traversal in these cases.

An intra-aortic balloon pump is inserted prophylactically in several cases to stabilize hemodynamics.^48^^,^^62^^,^^64^^,^^65^ Transseptal puncture can be performed with a standard needle, an electrified 0.035-inch wire, or a VersaCross RF wire. The Agilis NxT steerable introducer is then used to direct an electrified 0.014-inch or 0.035-inch wire, or a VersaCross wire, toward the base of the A2 segment of the anterior mitral leaflet. After successful traversal, the leaflet is dilated first with a 3- to 6-mm balloon, and then with a 12- to 14-mm balloon; the leaflet is not lacerated at this stage. Following enlargement of the septum with the same 12- to 14-mm balloon, the THV is advanced through the fenestration. During valve deployment, the leaflet is lacerated, eliminating the risk of LVOT obstruction. More recently, some operators have favored devices that can be used for both the septum and the leaflet, for example using the same 0.035-inch or VersaCross wire to cross both structures,^39^^,^^48^^,^^65^ and using the same 12- to 14-mm balloon to dilate both the septum and the leaflet^39^^,^^48^^,^^62^^,^^64^^,^^65^ (Table 5). Potential complications mainly include residual LVOT obstruction risk and injury to vascular and cardiac structures. A case of hemolysis post-BATMAN has also been reported, although it was more likely to be related to TMVR than the electrosurgical leaflet modification.^48^

### Transcaval access

Early-generation TAVR devices required large delivery systems that were often incompatible with transfemoral access in patients who had small or diseased iliofemoral arteries. For these patients, transcaval access offers an alternative route for large-bore device delivery by creating a controlled connection between the inferior vena cava and the abdominal aorta using transcatheter electrosurgery.

**Figure 5:**
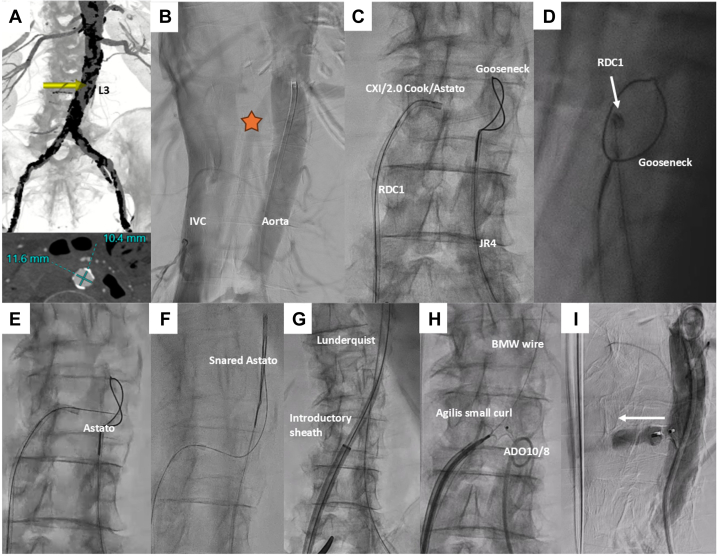
Transcaval TAVR (A) Preoperative computed tomography for evaluating feasibility and locating the traversal site. (B) Simultaneous aortogram and venogram to confirm the target puncture site. (C) Astato guidewire in the IVC and gooseneck snare in the aorta. (D) Orthogonal project to confirm the traversal site. (E) Astato wire traversal from the IVC to the aorta. (F) Astato wire snared in the aorta. (G) Delivery of TAVR sheath through the cavo-aortic access. (H) Closure of the cavo-aortic tract. (I) Aortogram to confirm satisfactory sealing of the cavo-aortic tract. ADO10/8 = amplatzer duct occluder 10/8; BMW = balance middleweight; IVC = inferior vena cava; JR4 = Judkins right 4; RDC1 = renal double-curve 1; other abbreviations as in Figure 1.

Transcaval TAVR is typically considered in patients who have no suitable femoral arterial access for TAVR procedures (Table 6). Pre-procedural computed tomography is essential for determining feasibility (Figure 5A). It is used to identify an optimal caval-aortic crossing site that has minimal aortic calcification and the shortest distance between the 2 vessels. A calcium-free window that is at least 2 mm larger than the planned sheath size is preferred. The crossing site also should be at least 10 mm away from major arterial branches, should not overlie bowel loops, and should maintain a safe margin from the femoral vein. It is also essential to develop bailout strategies, such as planning balloon sizes for aortic tamponade, which are generally oversized at 120% of the aorta at the crossing level.

**Table 6:** Common Tools for Transcaval TAVR

Electrosurgery generator, pencil, and forceps
GORE DRYSEAL Flex Introducer Sheath - 18/20/22 × 33 cm (Gore Medical); or Edwards eSheath+ Introducer Set (Edwards Lifesciences) for S3UR
Perclose ProGlide (Abbott) (or hemostatic suture)
BHW (Abbott) 0.014-inch 300 cm
Astato XS 20 300 cm 0.014-inch wire
NaviCross Angled-tip Support Catheter 4F 135 cm (Terumo)
Guiding catheter: 6F JR4 coronary length 100 cm; 6F/7F LIMA Renal Guide
Armada 35 Balloon Dilatation Catheter - 0.0350-inch (Abbott)
Amplatzer sizing balloon II - 20/27/40 mm (Abbott)
Snare catheter - gooseneck (20/25 mm; 5 mm larger than aortic diameter at target site)
Lunderquist Extra-Stiff wire 0.035-inch 260-cm - straight or single-curve (Cook Medical)
ADO-1 Amplatzer Duct Occluder - 10/8 (Abbott)
Covered stent - VIABHAN (Gore Medical)/ Endologix (Endologix)/ TriVascular (Boston Scientific)

TAVR = transcatheter aortic valve replacement.

During the procedure, orthogonal projections are identified and simultaneous aortogram and venogram are performed to confirm the target puncture site. A gooseneck snare is positioned in the aorta at the intended crossing level (Figures 5B to 5D). A crossing system, which includes a stiff guidewire within a wire converter and a support catheter, is advanced through the inferior vena cava to the crossing site. The guidewire is connected to a unipolar electrosurgery pencil using forceps to enable electrosurgical puncture of the vessel walls to achieve mid-point crossing (Figure 5E). Once the guidewire enters the aorta and is captured by the snare (Figure 5F), a wire exchange is performed, followed by serial dilatation of the tract and advancement of the TAVR delivery system (Figure 5F). After completion of the TAVR procedure, the aortic wall is then closed using nitinol occluder (Figures 5G and 5H). Potential complications mainly include bleeding and vascular complications.^66^, ^67^, ^68^

Transcaval procedures have been demonstrated to be a safe and effective route for performing TAVR^7^^,^^68^, ^69^, ^70^ and were introduced to the Asia-Pacific region in 2022^56^^,^^71^^,^^72^ (Table 7). Despite its potential benefits, transcaval access remains uncommon in the region, partly because of the limited experience in the region for the procedure. Other alternative vascular access, such as subclavian, transcarotid, and transaortic remain more common.

**Table 7:** Transcaval TAVR Outcome

Trial/Registry	No. of Patients	Traversal Success, %	Closure Success, %	Technical/ Procedural/ Clinical Success, %	VARC-2 Major Vascular Complication	Ref. #
Transcaval TAVR FIM	19	100	100	100	31.6	^7^
Transcaval TAVR IDE trial	100	99.0	99.0	98.0	13.0	^68^^,^^69^
European Transcaval TAVR Registry	50	100	98.0	94	14.0	^70^

Abbreviations as in Tables 2 and 6.

## Challenges and Future Development in the Asia-Pacific

The expansion of the transformative techniques in the Asia-Pacific region is partly limited by technical skills and tool accessibility barriers. The establishment of the Asia-Pacific Electrosurgery Working Group marks a significant step toward addressing these challenges through enhanced regional collaboration, training, and research.

### Technical skills barrier

One of the primary obstacles is the limited number of experienced operators in transcatheter electrosurgery. Electrosurgery remains a technically demanding skillset in structural interventions whereby on-site hands-on proctoring is crucial to ensure success at least in initial cases. To bridge this gap, regional unofficial proctorship programs are being developed to foster skill enhancement and proficiency. Because of the lack of official industry supports, proctoring often occurs as a peer-to-peer arrangement. Since 2022, leading conferences in the Asia-Pacific region have integrated dedicated transcatheter electrosurgery programs into their agendas. These sessions provide participants with hands-on experience using bench models, enabling them to learn the step-by-step process of the procedures. Furthermore, live case streaming from international renowned centers pioneering in electrosurgery have proven invaluable in demonstrating the practical application and nuances of the technique.

Despite these advances, some techniques, such as the CLEFT Electrosurgical Mitral Commissurotomy (electrosurgical laceration of rheumatic mitral valve leaflet), which may potentially be useful for patients in the Asia-Pacific region owing to the relatively high prevalence of chronic rheumatic heart disease, and ELASTA-Clip (electrosurgical laceration of the antero-posterior mitral tissue bridge),^73^ have yet to be introduced to the Asia-Pacific region.^8^, ^9^, ^10^ Given these are evidently a most difficult subset among electrosurgery procedures, the involvement of international proctors will be essential for effective knowledge transfer. It is hoped that some in the Asia-Pacific region will be able to train at international centers of excellence and return to become regional proctors. Equally important is the opportunity for fellows to participate in fellowships in expert centers abroad or attend specialized hands-on workshops, which would further augment their expertise and confidence.

### Tool access barrier

The catheters and tools that are commonly used for transcatheter electrosurgery are listed in Tables 1, 3, and 6. The development and introduction of specialized tools for transcatheter electrosurgery represent another critical frontier. At the time of writing, dedicated devices for transcatheter electrosurgery such as the TELLTALE and ShortCut systems are not yet available in the Asia-Pacific region.^35^ In certain regions, tools commonly used for transcatheter electrosurgery such as RF wires are not yet available in some Asia-Pacific countries. This lack of dedicated tools reflects the interplay of manufacturer marketing priorities, regional regulatory requirements, and other contributing factors. Moreover, some patients may find it challenging to bear the additional expense of dedicated devices. The absence of these specialized devices limits the scope of procedural innovation and efficiency. Therefore, facilitating the introduction of such tools into the region, along with nurturing local innovations in device technology, should be prioritized. This endeavor will require close collaboration between clinical experts and engineering professionals within both academia and industry, to ensure that the evolving needs of transcatheter electrosurgery are met by cutting-edge technological solutions.

## Collaborative Research

Research remains a critical area requiring attention. To date, there is no comprehensive Asia-Pacific registry dedicated to transcatheter electrosurgeries, which is crucial for summarizing regional experience and evaluating both safety and efficacy. The experiences documented in similar registries and prospective studies from the United States and Europe provide a valuable framework, underscoring the importance of establishing a similar repository in the Asia-Pacific region.^3^^,^^6^^,^^30^ Such a collaborative network would not only facilitate the collection of real-world data but also drive collaborative efforts in developing innovative techniques through preclinical models, followed by first-in-human trials and larger prospective studies.

## Conclusions

Transcatheter electrosurgery has emerged as a transformative technique in structural heart interventions, yet its adoption across the Asia-Pacific region remains in its infancy. The future development of transcatheter electrosurgery in the region hinges on a comprehensive strategy that integrates robust training and pioneering research. By uniting these efforts, the Asia-Pacific community can overcome current challenges, accelerate the adoption of advanced techniques, and set new standards in the practice of transcatheter electrosurgery.

## Funding Support and Author Disclosures

Dr Poon is a consultant for Anteris and is a proctor/speakers bureau for Edwards Lifesciences/Abbott. Dr So is a physician proctor for Abbott Structural Heart, Boston Scientific, Edwards Lifesciences, and Medtronic; and serves as consultant for Venus Medtech and Jenscare. Dr Yin is proctor and advisor for Medtronic and Edwards Lifesciences and receives consulting fees and honoraria. Dr Hayashida is clinical proctor for Edwards Lifesciences, Medtronic, and Abbot. Dr Ohno is a clinical proctor of transcatheter aortic valve implantation (TAVI) for Medtronic and a clinical proctor of TAVI and transcatheter edge-to-edge repair for Abbott Medical and has received speaker fees from Medtronic and Abbott Medical. Dr Ho received speaker fees from Edwards Lifesciences, Abbott Vascular, and Medtronic. Dr Kaneko serves as an advisory board member for Edwards Lifesciences, Abbott, 4C Medical, and Anteris; and a consultant for Medtronic. Dr Tang has received speakers honoraria and served as a physician proctor, consultant, advisory board member, TAVR publications committee member, RESTORE study steering and screening committee member, APOLLO trial screening committee member, and IMPACT MR steering committee member for Medtronic; has received speakers honoraria and served as a physician proctor, consultant, advisory board member, ENVISION trial screening committee member, and TRILUMINATE trial anatomic eligibility and publications committee member for Abbott Structural Heart; has served as an advisory board member for Boston Scientific; a consultant for Shockwave Medical, Anteris, Philips, Edwards Lifesciences, Peija Medical, and Shenqi Medical Technology; and has received speakers honoraria from Siemens Healthineers. Dr Khan received consulting/proctoring fees from Abbott, Edwards Lifesciences, and Medtronic; has equity in Transmural systems and Cuspa Medical; and is co-inventor on patents, assigned to the National Institutes of Health, on leaflet modification devices. All other authors have reported that they have no relationships relevant to the contents of this paper to disclose.
